# Integrating Computational Modeling and Experiments for the Additive Manufacturing of Copper-Based Antibacterial Coatings on 304SS Surface

**DOI:** 10.1021/acsphyschemau.5c00123

**Published:** 2025-12-09

**Authors:** Valentin Romanovski, Nickolay Sdobnyakov, Andrey Kolosov, Kseniya Savina, Mohammad Sharifian Gh, Nikita Nepsha, Denis Sokolov, Saravana Kumar M., Abhijit Bhowmik, Dmitry Moskovskikh, Marcos M. Pires, Elena Romanovskaia

**Affiliations:** † Department of Materials Science and Engineering, 2358 University of Virginia, Charlottesville, Virginia 22904, United States; ‡ Department of General Physics, 64970 Tver State University, Tver 170002, Russia; § Department of Chemistry, 2358 University of Virginia, Charlottesville, Virginia 22904, United States; ∥ Graduate Institute of Manufacturing Technology, 34877 National Taipei University of Technology, Taipei 10608, Taiwan; ⊥ Department of Additive Manufacturing, Mechanical Engineering, 487618 SIMATS, Saveetha Institute of Medical and Technical Sciences, Thandalam, Chennai 602105, India; # Division of Research and Development, Lovely Professional University, Phagwara, Punjab 144411, India; ∇ Science and Research Centre of Functional Nano-Ceramics, National University of Science and Technology “MISIS”, Lenin Av. 4, Moscow 119049, Russia

**Keywords:** 3D printing, antibacterial coatings, copper deposition, laser powder bed fusion, molecular dynamics simulation

## Abstract

The development of antibacterial coatings is very important for reducing pathogenic microorganisms on frequently touched surfaces. This study explores the formation of copper-based antibacterial coatings on 304 stainless steel using laser powder bed fusion (L-PBF) and integrates molecular dynamics (MD) simulations to analyze the melting and coalescence processes at the nanoscale. Experimental results showed heterogeneous copper distribution in the melting pool, with Cu-rich regions reaching up to 69 at. %. SEM-EDS analysis confirmed localized phase separation due to rapid solidification and Marangoni convection. MD simulations of Cu-304SS nanoparticles demonstrated significant copper surface segregation at 1600 K, validating experimental observations. The antibacterial efficacy of the coatings was assessed against *Escherichia coli* and *Acinetobacter baumannii*. Results showed complete bacterial inactivation within 1 h of exposure. These findings provide insights into optimizing L-PBF parameters for creating durable and efficient self-disinfecting surfaces.

## Graphical abstract

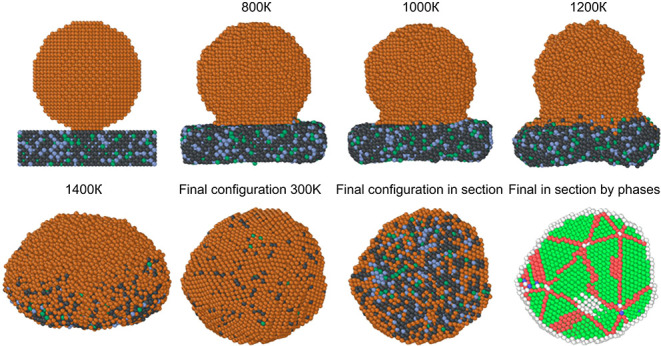

## 1 Introduction

Inactivation of microorganisms on surfaces and in water plays an important role in ensuring human health and well-being,^1–3^ as infectious diseases transmitted through contact surfaces and contaminated water remain one of the leading threats to public health.^4,5^ According to the World Health Organization (WHO), millions of people suffer from diseases caused by pathogenic microorganisms in drinking water and on surfaces every year, which emphasizes the need to develop effective disinfection methods. This problem is directly related to the implementation of the UN Sustainable Development Goals (SDGs), especially Goal No. 6 “Clean Water and Sanitation” and Goal No. 3 “Health and Well-being”. These goals are aimed at ensuring safe access to drinking water and improving sanitation, which requires the introduction of modern technologies for disinfection and preventing the spread of infections. In the context of the global fight against infections, the development of antibacterial coatings for surfaces that can effectively prevent the growth and spread of pathogens is of particular relevance. Such coatings are an important tool for reducing the risk of infection in healthcare facilities,^6^ public places, and the domestic environment.^7^ Effective antimicrobial coatings can significantly reduce the risk of pathogen transmission, making their implementation an important element of modern sanitary practices.

Copper, due to its pronounced antimicrobial properties, is a promising material for such applications. It is capable of inactivating a wide range of microorganisms, including bacteria and viruses, which makes it especially valuable for use on surfaces subject to frequent contact. However, traditional methods of applying copper coatings, such as chemical vapor deposition, electroplating, and vacuum deposition, have their limitations. They require complex equipment, expensive materials, or are not flexible enough in terms of structural control. For example, a study^8^ showed that traditional electroplated copper coatings on steel exhibit limited durability in high-humidity conditions. Modern methods of 3D printing metals open up new possibilities in the creation of antibacterial coatings. Additive manufacturing allows not only to minimize material waste but also to achieve a high degree of control over the microstructure and topography of surfaces. The use of 3D printing for material modification is becoming a promising direction due to the combination of precision, speed, and the ability to create complex geometries. Most studies are devoted to various modifications of already 3D-printed substrates,^9,10^ or printing a complete sample with copper or zinc additives.^11^ There is virtually no data on 3D-printed modification of substrates that allows preserving the mechanical and other properties of the original material. This approach also saves antibacterial material since it is used exclusively to create the coating. This, in turn, makes it possible to modify existing surfaces. This topic is a promising and little-studied area in materials science. One of the advantages of such coatings is the minimization of the use of liquid organic (for example, glutaraldehyde with a pH of 1% solution of about 4.1) or widely used chlorine-containing disinfectants (with a pH of working solutions above 9) or the use of ozone^12,13^ causing severe corrosion of the treated metal surfaces,^14,15^ as well as a negative impact on the environment during disposal of spent solutions.^16^ Among the promising materials for printing, silver,^17,18^ copper,^18^ and zinc^18,19^ can be considered.

The molecular dynamics (MD) method is a powerful tool for modeling processes at the atomic level. It allows analyzing thermal-mechanical interactions, crystallization dynamics, and alloying processes in metals. The application of MD modeling in this study is focused on the behavior of the melting zone during 3D printing of copper on stainless steel. This makes it possible to predict the properties of the resulting coating, including its adhesion, mechanical, and antimicrobial characteristics. The initial data for modeling, such as temperature and elemental parameters, were obtained from experiments, which ensures high accuracy of calculations.

The novelty of the approach proposed in the article lies in the combined use of experimental and computational methods for the development of a highly effective antibacterial coating. Our method demonstrates how 3D printing can be adapted to create antimicrobial surfaces with improved performance characteristics. The advantages include the possibility of local surface modification, flexibility in adjusting process parameters, and improved coating adhesion. Promising areas of application of such coatings include medical equipment, contact surfaces in public places, elements of transport infrastructure, and the food industry. In addition, this approach can be used to create coatings with other functional properties, such as corrosion resistance or improved mechanical properties, which opens up wide possibilities for further research and implementation.

The objectives of the research were 1) an experimental study of copper distribution in printed coatings, analysis of phase composition and microstructure using the SEM-EDS method, to identify patterns of copper segregation and mechanisms of interaction with a stainless steel substrate on a molecular level; 2) identification of patterns of structure formation during thermal interaction (coalescence) of copper and 304 steel at the nanolevel by predictive atomistic modeling of the coalescence process of nanoparticles of different configurations (two spherical nanoparticles and a spherical nanoparticle on a rectangular nanoplate).

## 2 Materials and Methods

### 2.1 Materials

Hot rolled 304 stainless steel (McMaster, USA) was used as the base for 3D printing. Cu powder of 99.6% purity with particle size distribution of 15–45 μm (EOS, USA) was used for coating.

### 2.2 Method of Copper Deposition

SLM125 laser deposition system (Nikon SLM Solutions Group AG, USA) was used for copper deposition. The L-PBF system uses an IPG Photonics fiber laser with a wavelength of 1070 nm and a spot size of 60.8 μm. Printing was performed in an argon environment with an oxygen content of less than 100 ppm. The process parameters used for printing Cu on 304 stainless steel are 300 W 600 mm/s at a layer thickness of 50 μm. Two samples were obtained with a step between lines in 500 and 800 μm.

### 2.3 Samples Analysis

The surface morphology of the sample was examined using a Hirox RH-8800 optical microscope (USA). A Quanta 650 scanning electron microscope (USA) equipped with an EDS probe was utilized for elemental analysis of the surface and the cross-sectioned sample.

The phase composition of the modified 304SS surface was analyzed using X-ray diffraction with an Empyrean diffractometer from Malvern-Panalytical (USA).

### 2.4 Molecular Dynamic Simulations

The modeling of the coalescence process of steel and copper alloy was performed using the molecular dynamics (MD) method. Alternative initial configurations were chosen as objects of study: two spherical nanoparticles in contact with each other, as well as a spherical nanoparticle on a rectangular nanoplate (simulating the real 3D printing process). Two nanoparticles, one of which was copper, and the second was a steel alloy (SS304) of different configurations: with a total number of atoms of 2000 (2 spherical particles of 2.5 nm), 5000 (2 spherical particles of 3.7 nm), 10000 (2 spherical particles of 9.8 nm), 20000 (2 particles of 19.1 nm). The ratio of the steel nanoparticle components corresponded to Area 304 in Table 2. The second configuration with a total number of 16,000 atoms consisted of a spherical copper nanoparticle (13.8 nm) and a rectangular Fe_5680_-Cr_1600_-Ni_720_ nanoplate (8 × 8 × 2 nm in size). Such simple models of initial configurations obviously fully describe the processes of structure formation during thermal sintering of copper and 304 steel. It is obvious that it is the atomistic modeling that can play a prognostic role, and comparison of the obtained results with experimental elemental maps allows one to control the process of structure formation. Modeling of the coalescence process was carried out in the temperature range from 300 to 1600 K, first heating and then cooling occurred. Before each calculation, the nanoparticles underwent a relaxation process for 15 ps. In the process of MD modeling, the same heating and cooling rate of 0.5 K/ps was used. At present, the use of such rates in modeling thermally induced structural transformations is fully justified, since ultrafast cooling technologies have been developed by now.^20^ It is obvious that periodic boundary conditions were also used in the modeling process.

The MDSym and LAMMPS software were used, using the tight-binding potential (TBP), the parameters of which are given in Table 1.^21,22^ To calculate the TBP cross parameters, the Lorentz–Berthelot rule was used, tested not only for binary nanoparticles,^23–26^ but also for multicomponent ones.^27–29^ Also, the classical Nosé–Hoover thermostat was used in the LAMMPS software, and the soft stochastic thermostat^30^ was used in MDSym, which is a Nosé–Hoover thermostat with the addition of random noise to improve ergodicity. Despite its stochastic nature, the thermostat weakly affects the physical dynamics measured by the perturbation of time autocorrelation functions. Additionally, in order to more accurately determine the phase transition temperature, an analysis of the presence of crystalline phases (*fcc*, *hcp*, *bcc*, IR nuclei) was carried out by matching polyhedral templates using the OVITO program.^31^ This method is based on the successive superposition of the local neighborhood of the atom on each of the ideal geometric structures, which allows for the precise determination of the type of crystalline phase. The value 0.155 was used for the trimming parameter of the Root-mean-square deviation value used in the polyhedral template matching method.

**Table 1:** Parameters of the Potential

Metal	*A*, eV	ζ, eV	*p*	*q*	*r*_0_, Å	*r*_cut_, Å
Cu–Cu	0.0855	1.224	10.96	2.278	2.556	7.55
Cu–Ni	0.0567	1.1444	13.9795	1.7335	2.5239	7.55
Cu–Cr	0.059	1.161	12.0726	1.5886	2.5271	7.55
Cu–Fe	0.1006	1.3737	10.8607	2.158	2.5192	7.55
Ni–Ni	0.0376	1.07	16.999	1.189	2.4918	7.55
Ni–Cr	0.0391	1.0855	15.0921	1.0442	2.495	7.55
Ni–Fe	0.0667	1.2844	13.8802	1.6135	2.4871	7.55
Cr–Cr	0.0407	1.1012	13.1852	0.8993	2.4981	7.55
Cr–Fe	0.0694	1.303	11.9733	1.4686	2.4903	7.55
Fe–Fe	0.1184	1.5418	10.7613	2.0379	2.4824	7.55

### 2.5 Antimicrobial Properties

The antibacterial activity of the substrates was tested against two bacterial strains: Gram-negative *Escherichia coli* (*E. coli* BW25113) and *Acinetobacter baumannii* (NCBI 2208). A single bacterial colony was grown aerobically at 37 °C with 250 rpm shaking in Luria Broth medium for 16–18 h. The bacterial cultures were then diluted 100,000 times in phosphate-buffered saline (pH 7.3). A 20 μL drop of each diluted bacterial suspension was applied to the substrate and incubated at room temperature for 1 h. To minimize evaporation, a water bath was used. After incubation, the samples were transferred to Luria Broth agar plates and incubated at 37 °C for 24 h. Colony growth was visualized and documented using BIO-RAD imaging equipment.

## 3 Results and Discussion

### 3.1 Samples Characterization

The profilometric image of the sample shows a three-dimensional surface profile characterized by intersecting printed lines at intervals of approximately 500 and 800 μm (Figure 1). The uniformity of the printed lines can be noted, with a relatively constant height along their entire length, except at intersections where material convergence is evident. The profilometric data highlights the accuracy of the printing process and also identifies areas for potential optimization, such as ensuring a uniform copper layer height distribution.

**Figure 1:**
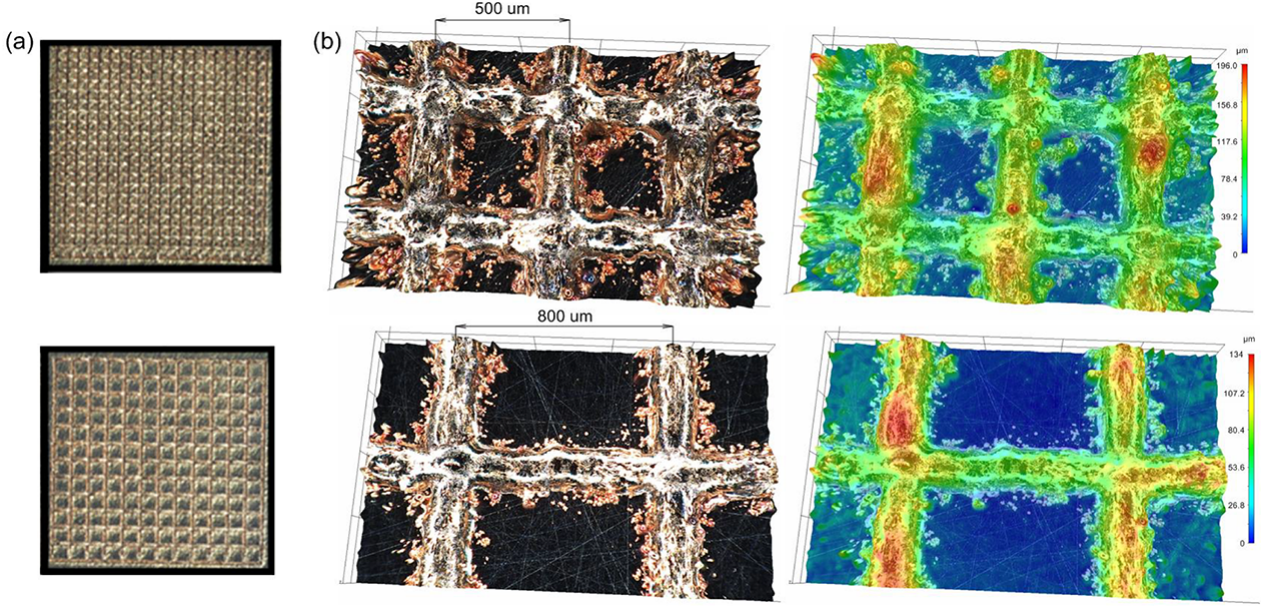
Sample overview (a), and 3D surface profilometry of a sample (500 μm top and 800 μm bottom) with intersecting printed lines (b).

The SEM-EDS analysis of the sample revealed a unique microstructural evolution within the melting pool during the 3D printing process (Figure 2). A cross-section of the printed coating reveals a clearly defined fusion zone in the steel, approximately 175 ± 41 μm deep and 151 ± 3 μm wide. A protruding copper layer, 60 ± 8 μm high, forms above the original 304SS surface, indicating stable molten pool formation and good adhesion between the copper and the substrate. The selected laser printing parameters ensured stable formation of the molten pool and prevented the formation of cracks and pores within its volume. Spherical zones with varying copper concentrations were observed (Figure 2), indicating a dynamic mixing of molten stainless steel and copper particles. These zones, formed due to Marangoni convection and rapid solidification,^32–34^ exhibit a distinct pattern of copper-rich and copper-depleted regions (Table 2). Copper-enriched areas likely result from localized segregation during cooling, where the difference in melting points and densities between copper and steel plays a crucial role.

**Figure 2:**
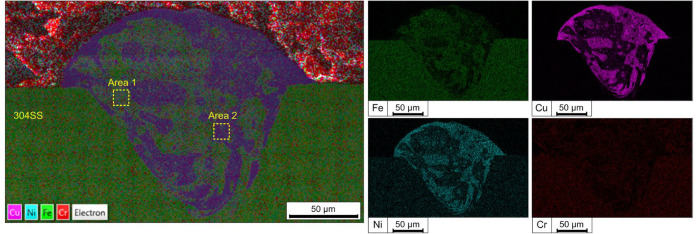
SEM-EDS mapping of printed Cu on SS304 in cross-section.

**Table 2:** Elemental Composition of the Steel Substrate and Selected Areas in the Melted Pool (Based on Figure 2), at. %

Area	Fe	Cr	Ni	Cu
304	71.8	20.0	8.2	0.0
Area 1	55.3	14.8	6.1	23.7
Area 2	20.5	6.1	4.4	69.0

The EDS mapping further confirmed the heterogeneous distribution of copper within the matrix, with bright spots corresponding to higher copper concentrations and darker regions representing a predominantly steel matrix. This phenomenon aligns with previously reported studies, where similar immiscible alloy systems demonstrated liquid-phase separation during solidification. Such microstructural characteristics contribute to unique mechanical and antimicrobial properties of the resulting surface, providing insights into optimizing printing parameters for tailored performance.

Figure 3 presents the XRD pattern of the surface of the obtained sample. The diffraction peaks correspond to the primary phases of austenitic stainless steel (*fcc*) and copper (*fcc*), indicating the coexistence of both Cu and 304SS in the printed coating. The presence of distinct Cu peaks suggests partial segregation of copper within the material rather than complete dissolution in the steel matrix. No intermediate Fe–Cu intermetallic phases were detected, which is consistent with the limited solubility of copper in iron under rapid solidification conditions.^35–37^ The relative intensity of the Cu peaks indicates a heterogeneous distribution of copper, aligning with SEM-EDS observations. Additionally, slight peak shifts in the iron phase suggest residual stresses induced by the laser melting process. The XRD data confirm that the printed coating maintains a predominantly biphasic structure,^38,39^ with copper retained in a separate phase, which is critical for its antibacterial functionality. These findings correlate well with molecular dynamics (MD) simulations (see next subsection), supporting the observed microstructural features and phase distribution in the coating.

**Figure 3:**
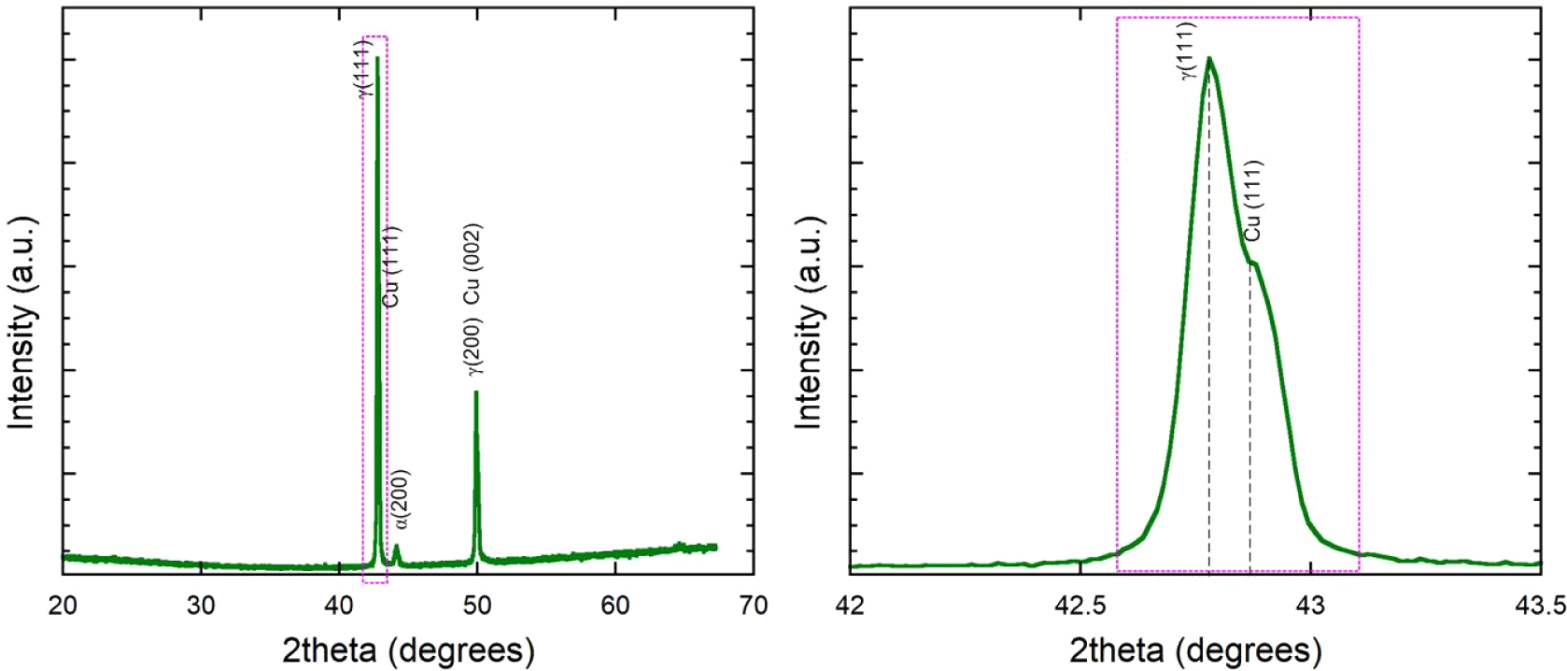
XRD pattern of the obtained surface.

### 3.2 Molecular Dynamic Simulations

The main information about the melting and crystallization processes, as well as the process of their coalescence with qualitative changes in the configuration of nanoparticles, can be obtained from the analysis of the caloric dependences of the potential part of the specific internal energy U (per atom). In Figure 4a–d, such typical (corresponding to a single calculation from a series of experiments) dependences are given for a system with an initial configuration corresponding to two spherical nanoparticles of copper and steel. As the system size increases, the absolute value of the specific potential energy increases both at the initial temperature and at the temperature corresponding to the final configuration. Thus, it can be concluded that the nanosystem becomes more stable as the number of atoms in it increases. It should also be noted that the difference between the specific potential energies corresponding to the beginning and end of the simulation process decreases with increasing system size and is less than 0.008 eV/atom for a system size of 19.1 nm. For the case of an alternative initial configuration – a spherical nanoparticle on a rectangular nanoplate, the patterns described on the basis of the analysis of the caloric curves of the potential part of the specific internal energy do not change conceptually (Figure 6a). All dependences demonstrate the presence of hysteresis of the melting temperatures *T*_m_ and crystallization *T*_c_.^40,41^ It was confirmed that for both binary^26,42^ and multicomponent nanoparticles^27^− the melting and crystallization processes do not correspond to one strictly defined temperature but occur in a certain temperature range (Δ*T*_m_ = *T*_m_^f^ – *T*_m_^s^ and Δ*T*_c_ = *T*_c_^s^ – *T*_c_^f^, the indices s and f correspond to the beginning and end of the corresponding process). In this case, the value of this range depends not only on the size of the nanoparticles but also on the geometry of the system, the ratio of components, and possibly a number of other factors. Table 3 presents numerical estimates of the temperatures of the beginning and end of phase transitions and the width of the corresponding temperature range, which allows us to trace their size dependence. As expected,^43^ for the four-component nanoparticle (Cu–Fe–Ni-Co) a size effect is recorded for both the melting temperature and the crystallization temperature, although in this case the size effect is weaker.^40,44^ In this case, nonlinear behavior is characteristic of the width of the temperature range in which melting and crystallization occur. Up to a certain critical size of the system, an increase is observed (for both melting and crystallization) and then a decrease, which is obviously due to the fact that in the macroscopic limit the phase transition processes (melting and crystallization) occur at a fixed temperature.

**Figure 4:**
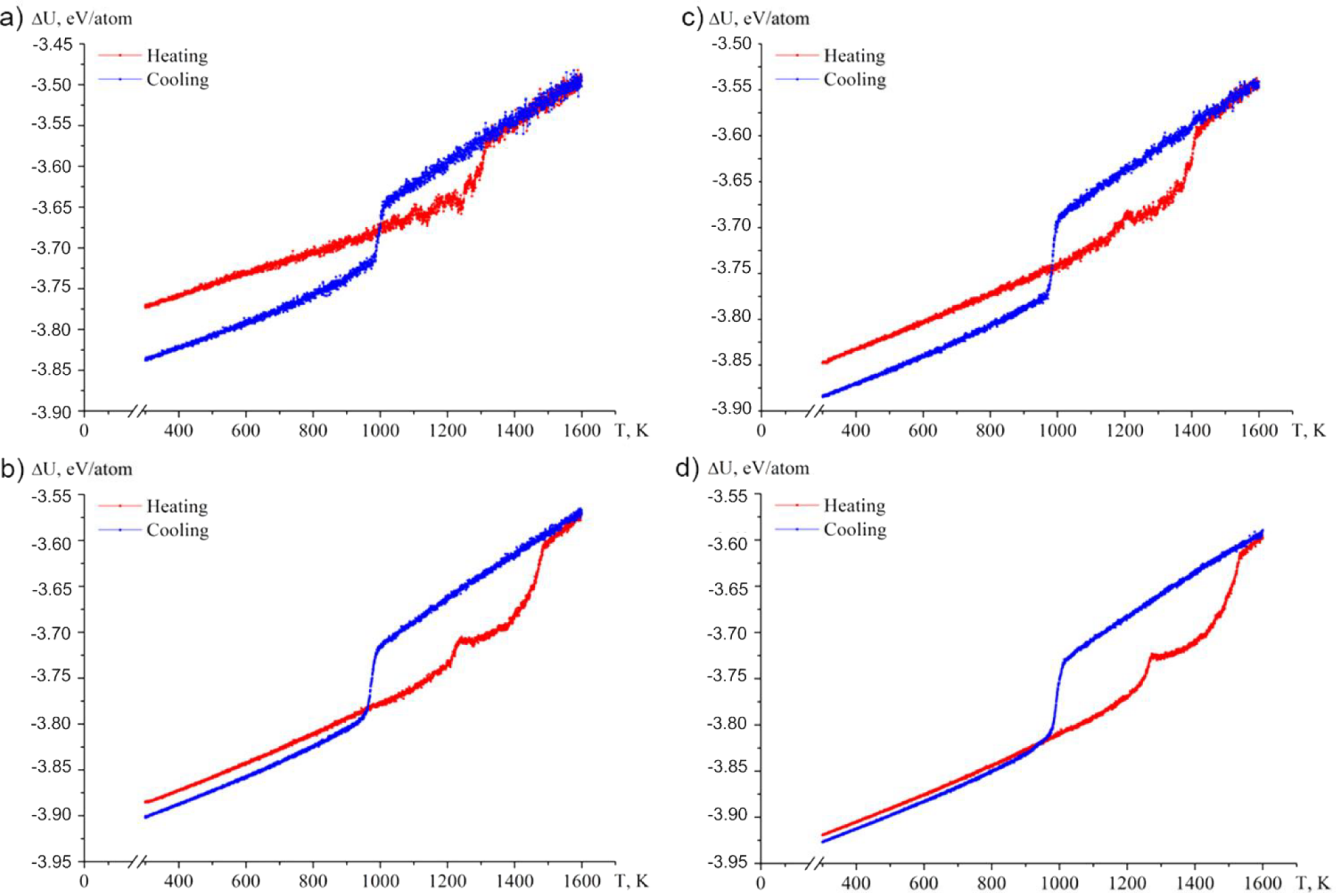
(a) Caloric curves of the coalescence process and subsequent crystallization for two nanoparticles Cu_1000_ and Fe_710_-Cr_200_-Ni_90_ (2.5 nm); (b) Cu_2500_ and Fe_1775_-Cr_500_-Ni_225_ (3.7 nm); (c) Cu_5000_ and Fe_3550_-Cr_1000_-Ni_450_ (9.8 nm); (d) Cu_10000_ and Fe_7100_-Cr_2000_-Ni_900_ (19.1 nm).

**Table 3:** Hysteresis Parameters of Melting and Crystallization Processes Obtained on the Basis of the Analysis of Caloric Curves of the Potential Part of the Specific Internal Energy U (Data Averaged Over a Series of Calculations)

Composition	$T_{c}^{s}$ , K	$T_{c}^{f}$ , K	$\Delta T_{c}$ , K	$T_{m}^{s}$ , K	$T_{m}^{f}$ , K	$\Delta T_{m}$ , K	$T_{m}^{\mathrm{coal}}$ , K
Cu_1000_ and Fe_710_-Cr_200_-Ni_90_	1005	985	20	1280	1320	40	1105
Cu_2500_ and Fe_1775_-Cr_500_-Ni_225_	1008	975	33	1375	1420	45	1210
Cu_5000_ and Fe_3550_-Cr_1000_-Ni_450_	1010	960	50	1390	1485	95	1240
Cu_8000_ and Fe_5680_-Cr_1600_-Ni_720_	1012	975	37	1400	1475	75	1245
Cu_10000_ and Fe_7100_-Cr_2000_-Ni_900_	1015	980	35	1480	1530	50	1275

The caloric curves corresponding to melting (Figures 4a–d and 6a) also show that there is a jump in the temperature range of 1100–1280 K (*T*_m_^coal^). This jump corresponds to the beginning of the copper melting process and the beginning of the coalescence process of two nanoparticles. The *T*_m_^coal^ value is also characterized by a size effect. Active diffusion and segregation processes contribute to the equalization or even decrease in the energy of the nanosystem with increasing temperature. This phenomenon is observed almost always when coalescence occurs after the melting process of one of the particles. It is worth noting that the melting and crystallization temperatures do not depend on the shape of the nanoparticles, only the size effect is observed for the thermodynamic characteristics (temperatures, heats of the corresponding phase transitions).^45–47^ The general form of the behavior of the dependence of the potential part of the specific internal energy on temperature (hysteresis) remains the same in all cases. Figure 5 allows to visually trace the segregation processes during coalescence of copper and steel nanoparticles; in fact, these images are a kind of analogue of experimental EDS mapping. Using the Ovito software, it can be established that in all final configurations of nanoparticles, the *fcc* structure with hcp elements predominates. In this case, the hcp structure is represented either by local zones or by alternating planes (Figure 5). With an increase in the size of the nanosystem, a transition to a more complex local structure is demonstrated, namely, a pattern of alternating zones of the local *fcc* structure and hcp planes. As can be seen from Figure 5, in general, for the system represented by a spherical nanoparticle on a rectangular nanoplate, the stages of structure formation after the completion of the coalescence process fully correlate with the patterns demonstrated by a system consisting of two spherical nanoparticles (Figure 5). The data presented in Table 4 provide the calculated values of the degree of crystallinity for the configurations under consideration. From Table 4, it can be seen that for the given configurations, the degree of crystallinity increases with increasing size, which also indicates enhanced stability of the nanoparticles. The variation of the *bcc* local structure is directly related to defects; these regions appear at the boundaries between *hcp* and *fcc* local structures. In this case, it is the *fcc* local phase that dominates.

**Figure 5:**
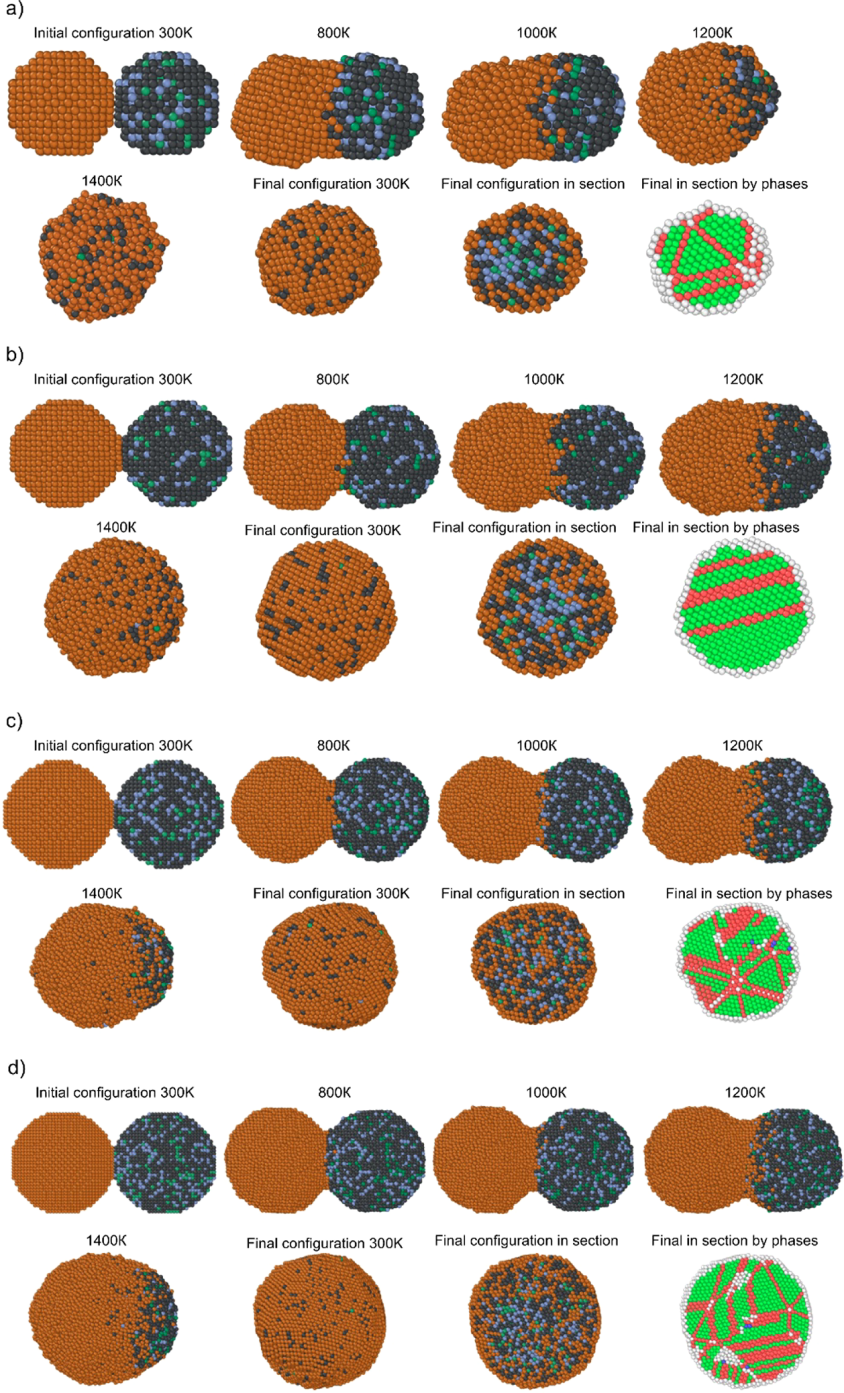
(a) Cu_1000_ and Fe_710_-Cr_200_-Ni_90_ nanosystem (2.5 nm); (b) Cu_2500_ and Fe_1775_-Cr_500_-Ni_225_ nanosystem (3.7 nm); (c) Cu_5000_ and Fe_3550_-Cr_1000_-Ni_450_ nanosystem (9.8 nm); Cu_10000_ and Fe_7100_-Cr_2000_-Ni_900_ nanosystem (19.1 nm); (d) Cu_10000_ and Fe_7100_-Cr_2000_-Ni_900_ nanosystem (19.1 nm). Brown atoms are copper, dark gray are iron, blue is chromium, and dark green is nickel. For phase distribution, light green atoms are *fcc* local environment, red ones are *hcp*, blue ones are *bcc*, and white ones are unrecognized.

**Table 4:** Distribution of Local Phases in Final Configurations^a^

Composition	*fcc*	*hcp*	*bcc*	Unrecognized	η, %
Cu_1000_ and Fe_710_-Cr_200_-Ni_90_	706	541	0	753	62.4
Cu_2500_ and Fe_1775_–Cr_500_-Ni_225_	2673	1002	4	1321	73.6
Cu_5000_ and Fe_3550_-Cr_1000_-Ni_450_	4705	2605	176	2514	74.9
Cu_8000_ and Fe_5680_-Cr_1600_-Ni_720_	8917	3588	93	3402	78.7
Cu_10000_ and Fe_7100_-Cr_2000_-Ni_900_	9110	6503	90	4297	78.5

a Here, parameter η denotes the degree of crystallinity, i.e., the ratio of the number of atoms included in the recognized local phases to the total number of atoms (see Figures 5 and 6b).

During coalescence, active segregation of copper to the surface can be observed. Copper covers the entire nanoparticle even in the melt at 1600 K, allowing only single iron atoms to pass to the surface. Iron atoms are most often located in the near-surface zone, chromium atoms are closer to the core, and nickel atoms are distributed uniformly throughout the particle. This behavior is associated with factors^48^ that determine the patterns of surface segregation, in particular, the difference in the values of the surface energy of the components and the corresponding size effect.^47,49,50^ Thus, the concept^27^, is confirmed that the segregation behavior of multicomponent metallic nanoparticles allows subdividing the atoms of their components into three types: 1) atoms exhibiting a tendency to surface segregation; 2) atoms forming the core of the nanoparticle, as well as its peripheral regions; 3) atoms indifferent to segregation processes. However, the final structure cannot be called uniformly distributed. With increasing nanoparticle size, it becomes more noticeable (lower left inset Figures 5 and 6b) that there are copper-rich areas and iron-rich areas. This behavior agrees quite well with the experimental data obtained in this work (Figure 1).

**Figure 6:**
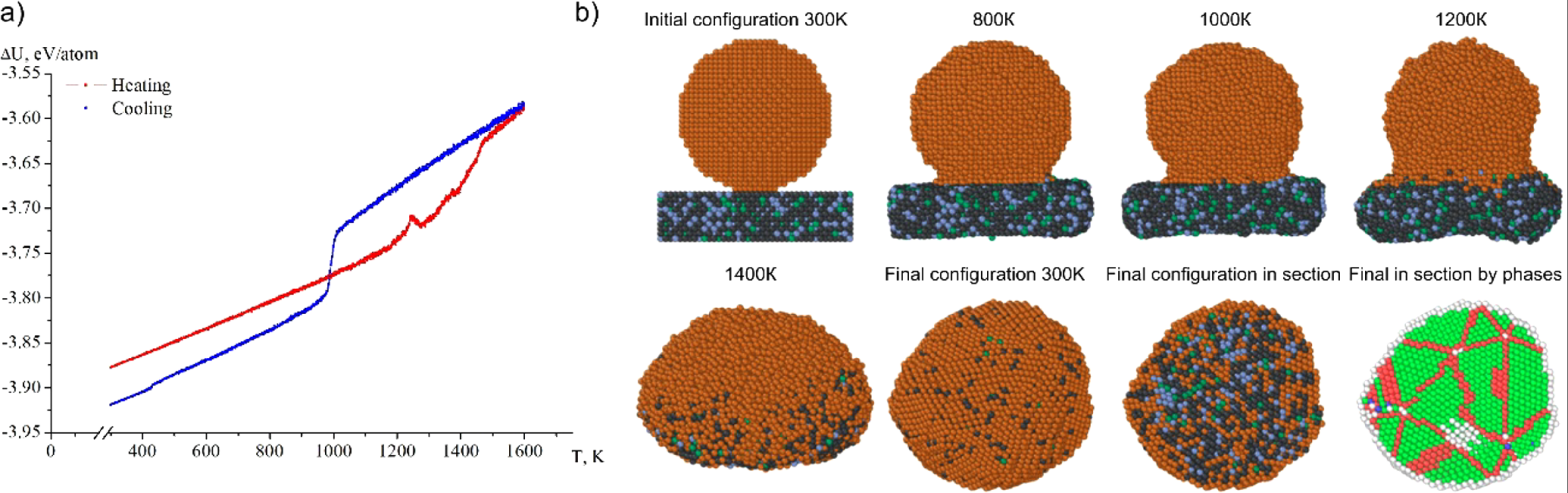
(a) Caloric curves of the coalescence process and subsequent crystallization for two nanoparticles Cu_8000_ and Fe_5680_-Cr_1600_-Ni_720_ (13.8 nm); (b) nanosystem Cu_8000_ and Fe_5680_-Cr_1600_-Ni_720_ (13.8 nm). Brown atoms are copper, dark gray are iron, blue is chromium, and dark green is nickel. For phase distribution, light green atoms are *fcc* lattice, red are *hcp*, blue are *bcc*, and white are unrecognized.

### 3.3 Antibacterial Properties

Figure 7a shows the results of inactivation of *E. coli* and *A. baumannii* on 304 and copper as a control and test sample, for 1 h at room temperature. The results show that the steel substrate did not exhibit significant antibacterial properties, and that the growth was comparable to that of untreated control cells. At the same time, copper and the test samples demonstrated significant antibacterial properties, with no colonies growing after 1 h of exposure. On the surface of the sample (Figure 7b), residual structures and fragments of microorganisms are visualized after their inactivation, confirming the effectiveness of the antibacterial coating.

**Figure 7:**
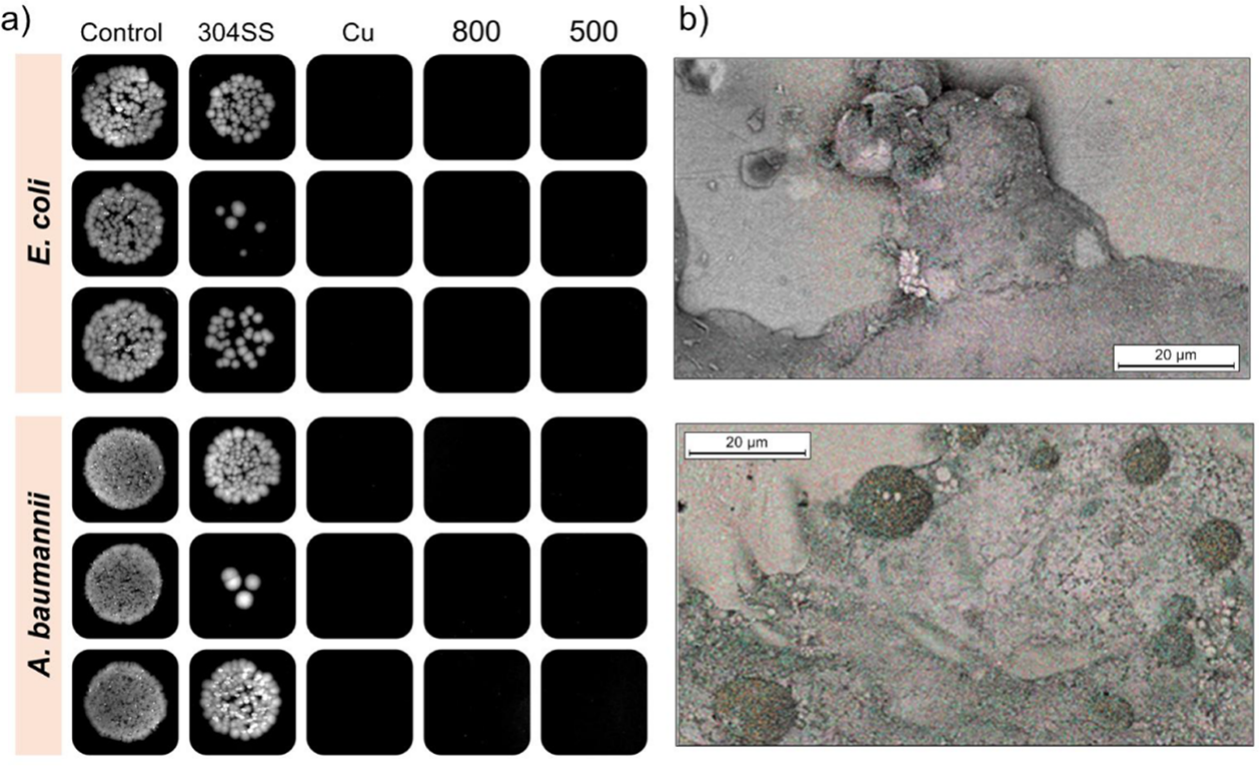
Antibacterial activity of the 304SS, Cu, and 304-Cu sample against *E. coli* and *A. baumannii* bacteria (a), and residual structures and fragments of microorganisms (b).

The mechanism of the antibacterial action of copper coatings is associated with a combination of processes involving the electrochemical release of Cu^+^/Cu^2+^ ions, direct damage to the cell wall, and the generation of reactive oxygen species. When microorganisms come into contact with the copper surface, copper dissolves and cations are released, which interact with membrane proteins and lipids, disrupting its integrity and function. Additionally, cathodic reactions on copper lead to the formation of peroxide and oxygen radicals, which increase oxidative stress and cause damage to nucleic acids and enzymes.^51^ As shown in our studies, the composition and stability of oxide films (Cu_2_O, CuO, Cu­(OH)_2_) regulate the rate of ion release, and the observed heterogeneous segregation of copper ensures the presence of localized Cu-rich regions that support a constant cation flow and the formation of ROS. The combined action of these factors explains the complete inactivation of *E. coli* and *A. baumannii* bacteria within 1 h.

The stability of the antimicrobial effect is indeed an important factor, and this aspect was examined in our previous studies.^51^ Specifically, it was shown that pure copper maintains high microbial inactivation efficiency; however, with prolonged exposure to moisture and disinfectant solutions, it quickly becomes coated with corrosion products, which can alter the kinetics of Cu^+^/Cu^2+^ ion release. Meanwhile, the Cu-30Ni alloy exhibits more stable behavior: it maintains a shiny surface and corrosion resistance even after multiple cleaning cycles, while its virus and bacterial inactivation efficiency decreases only slightly and remains comparable to that of pure copper. Thus, the long-term stability of the antimicrobial effect depends on the coating composition and operating conditions, opening up opportunities for further optimization of the materials and expanding their range of applications.

A promising direction for further research is to modify the proposed approach by alloying copper with other metals or altering the surface topography. As shown in recent studies, pure copper exhibits high antimicrobial activity, but is susceptible to intense corrosion and quickly loses its appearance when exposed to disinfectants and humid atmospheres. Meanwhile, the Cu-30Ni alloy demonstrates comparable microbial inactivation efficiency with significantly greater corrosion resistance and retention of a bright metallic luster.^51^ This combination of properties makes copper–nickel coatings attractive for use on high-contact surfaces, where not only antimicrobial properties are important but also durability, aesthetics, and stability under regular cleaning. Such modifications open up opportunities for expanding the technology’s application range, including medical equipment, transportation infrastructure, and public facilities.

## 4 Conclusion

Laser powder bed fusion (L-PBF) based synthesis of copper coatings exhibited a high deposition of copper on a surface of the 304 stainless-steel substrate, and it retained the structural integrity. By using SEM-EDS analysis the presence of heterogeneous copper-enriched and copper-deficient zones has been demonstrated, which are controlled via fast solidification and Marangoni convection. XRD characterization revealed a biphasic structure consisting of *fcc* Fe and *fcc* Cu, with no detectable intermetallic phases, indicating limited solubility of copper in the steel matrix. These results highlight the potential of L-PBF for creating durable and effective antibacterial coatings with controlled microstructure and composition.

Using the example of atomistic modeling of the coalescence process of nanoparticles of different configurations (two spherical nanoparticles and a spherical nanoparticle on a rectangular nanoplate), the patterns of structure formation during the interaction of copper and 304 steel at the nanoscale were revealed. First, it was found that the initial configuration does not affect the structure formation processes occurring after the coalescence process is complete. In this case, the size effect has an effect. Second, based on the analysis of the caloric dependences of the potential part of the specific internal energy for a four-component nanoparticle (Cu–Fe–Ni–Co), it is possible to identify the presence of hysteresis of melting and crystallization temperatures, the presence of a finite temperature range in which the corresponding phase transition occurs. In this case, the width of this interval depends on the size of the system nonlinearly (first it increases to a certain limiting value, then decreases and it is obvious that in the macroscopic limit (Δ*T*_m_ → 0 and Δ*T*_c_ → 0 ). As expected (a metal with a lower surface energy will segregate to the surface of the nanoparticle), copper atoms segregated to the surface of the nanoparticle. For the four-component nanosystem (Cu–Fe–Ni–Co), all other components except copper do not show an obvious tendency to form a core, and considering their mass fraction, they are distributed fairly uniformly throughout the entire volume of the nanoparticle except for the surface (1–2 monolayers), which is due to the close value of the surface energy of the components (Fe, Ni, Co) considering the size effect. Third, both the results of atomistic modeling of the nanosystem (Cu–Fe–Ni–Co) and the EDS mapping data predict the possibility of forming a structure unevenly distributed over the composition of the components, in particular, the formation of local areas enriched in copper and areas enriched in iron. In addition, it can be concluded that in the case of experimental production of alloys with a uniform distribution of components, then on scales of less than 20 nm the structure can be nonuniform in composition, which is explained by the processes of local structure formation at the nanolevel. Thus, the combined use of experimental methods for the synthesis of alloys, the study of their phase and elemental composition, including the construction of EDS mapping in combination with atomistic modeling is an effective tool for predicting and verifying the structure and structural transformations, changing the thermodynamic characteristics corresponding to melting and crystallization at the nanolevel.

## Data Availability

The data that support the findings of this study are available from the corresponding author upon reasonable request.
